# Identifying occupational carcinogens: an update from the IARC Monographs

**DOI:** 10.1136/oemed-2017-104944

**Published:** 2018-05-16

**Authors:** Dana Loomis, Neela Guha, Amy L Hall, Kurt Straif

**Affiliations:** IARC Monographs Programme, International Agency for Research on Cancer, Lyon, France

**Keywords:** cancer, occupational exposures, epidemiology

## Abstract

The recognition of occupational carcinogens is important for primary prevention, compensation and surveillance of exposed workers, as well as identifying causes of cancer in the general population. This study updates previously published lists of known occupational carcinogens while providing additional information on cancer type, exposure scenarios and routes, and discussing trends in the identification of carcinogens over time. Data were extracted from International Agency for Research on Cancer (IARC) Monographs covering the years 1971–2017, using specific criteria to ensure occupational relevance and provide high confidence in the causality of observed exposure-disease associations. Selected agents were substances, mixtures or types of radiation classified in IARC Group 1 with ‘sufficient evidence of carcinogenicity’ in humans from studies of exposed workers and evidence of occupational exposure documented in the pertinent monograph. The number of known occupational carcinogens has increased over time: 47 agents were identified as known occupational carcinogens in 2017 compared with 28 in 2004. These estimates are conservative and likely underestimate the number of carcinogenic agents present in workplaces. Exposure to these agents causes a wide range of cancers; cancers of the lung and other respiratory sites, followed by skin, account for the largest proportion. The dominant routes of exposure are inhalation and dermal contact. Important progress has been made in identifying occupational carcinogens; nevertheless, there is an ongoing need for research on the causes of work-related cancer. Most workplace exposures have not been evaluated for their carcinogenic potential due to inadequate epidemiologic evidence and a paucity of quantitative exposure data.

## Introduction

Historically, much of what was known about the causes of cancer was derived from studies of workers. Indeed, an observant 18th-century physician’s conclusion that cancer of the scrotum in young chimney sweeps was caused by their occupational exposure to soot, later found to contain polycyclic aromatic hydrocarbons,^1 2^ is often cited as the first clear identification of a carcinogen (eg, refs 3 4). With the notable exception of tobacco smoking, most of the other carcinogens that were recognised during the 19th to mid-20th centuries were discovered through similar observations.^5^ Even after several decades of intensive research beginning in the mid-20th century, nearly half of the ‘established human carcinogens’ listed in Doll and Peto’s seminal report on the avoidable causes of cancer were occupational in nature.^3^ These discoveries have been facilitated by characteristics of the work environment that allow cancer occurrence to be studied, notably well-defined populations that are exposed, often at high levels, to agents that can be quantitatively characterised. Analytical methods first developed to study occupational cancer have also contributed importantly to the development of modern epidemiology.^6^

Identifying occupational carcinogens is an important research endeavour with broad relevance to science and public health. Occupational exposure to carcinogens is a major cause of death and disability worldwide,^7^ with an estimated occurrence of 666 000 fatal work-related cancers annually.^8^ Knowledge of cancer hazards from occupational exposure supports prevention and surveillance activities, as well as compensation of exposed workers. However, creating a list of occupational carcinogens is not a trivial exercise, as there is neither a consensus definition of such agents nor a single, definitive source of all the relevant data. Doll and Peto^3^ provided a table of ‘established occupational causes of cancer,’ but did not specify the methodology by which they were identified. Some 20 years later, Siemiatycki and coauthors^9^ developed a list of ‘definite occupational carcinogens’, drawing on data from the *IARC Monographs on Carcinogenic Risks to Humans* published through 2003 and other sources. The International Agency for Research on Cancer (IARC) Monographs have been updated since then: more than 120 additional agents have been evaluated in 36 new volumes; furthermore, the methodology for evaluating the evidence base has been updated,^10 11^ and a re-evaluation of the agents classified as ‘carcinogenic to humans’ in the first 99 volumes has been completed with additional target organ sites identified in the process.^12^

Here we provide an updated listing of occupational carcinogens that includes data through volume 120 of the IARC Monographs corresponding to the years 1971–2017. We also provide additional information on tumour type, exposure scenarios and exposure routes, identify methodological challenges in compiling such a list from available data sources, and discuss trends in the identification of carcinogens over time.

## Methods

As a primary source of data, we used the *IARC Monographs on Carcinogenic Risks to Humans*, the world’s most comprehensive encyclopaedia of evaluations of carcinogenicity, comprising over 1000 entries.^13^ The review and evaluation methods used to develop the IARC Monographs are documented in the *IARC Monographs* Preamble.^10^

Briefly, agents are selected for review based on evidence of human exposure and published scientific data suggestive of carcinogenicity. For each agent evaluated, systematic reviews of the available scientific evidence concerning the carcinogenicity of the agent in humans and experimental animals are conducted by an international working group of independent experts. Each line of evidence is evaluated according to ordered categories that reflect the strength of the evidence of carcinogenicity. The highest category of ‘sufficient evidence of carcinogenicity’ in humans or animals means that a causal relationship between exposure to the agent and development of cancer has been established. For epidemiological data, ‘sufficient evidence of carcinogenicity’ is typically based on results from several well-designed, well-conducted studies where chance, bias and confounding could be ruled out with reasonable confidence; the conclusion is unlikely to be altered by future studies. Data on human exposure to the agent and toxicological data on pertinent mechanisms of carcinogenesis are also reviewed.

An overall evaluation integrating epidemiological and experimental data is derived according to a structured process that accounts for the strength of evidence for carcinogenicity in humans, animals and mechanistic evidence, most notably in exposed humans. Agents with ‘sufficient evidence of carcinogenicity’ in humans are assigned by default to the highest category, ‘carcinogenic to humans’ (IARC Group 1) whereas the categories of ‘probably’ (Group 2A) or ‘possibly’ (Group 2B) carcinogenic to humans, or ‘not classifiable as to its carcinogenicity to humans’ (Group 3) are assigned according to the combined strength of the human, animal and mechanistic evidence. Evaluations may be upgraded to a higher category when the evidence for a relevant mechanism of carcinogenesis is sufficiently strong. From the initiation of the IARC Monographs programme in 1971 to date, 119 agents have been classified in Group 1, 81 in Group 2A and 299 in Group 2B. These classifications refer to the strength of the evidence for a cancer hazard, rather than to the level of cancer risk.

### Definitions

In the absence of a consensus definition of an occupational carcinogen, we developed the following criteria:

1. The agent is a defined substance, a mixture, or a type or source of radiation.
2. The agent is classified in IARC Group 1 with ‘sufficient evidence of carcinogenicity’ in humans (to ensure that observed exposure-disease associations are causal).
3. ‘Sufficient evidence of carcinogenicity’ in humans is obtained entirely or in part from epidemiologic studies of exposed workers (to ensure that the carcinogen has documented occupational exposure); the occurrence of exposure in workers is documented in the pertinent monograph.

Evaluations based on an occupational title, industry or production process without specification of causal agents were also recorded, but were considered separately since they are qualitatively different from the other classes of agents and afford limited opportunities for prevention. Furthermore, such evaluations are time sensitive given that processes, materials and exposures change over time. Infectious agents and pharmaceutical preparations, including botanicals, hormones and antineoplastic agents, were effectively excluded because the pertinent monographs did not provide information indicating occupational exposure. These exclusions also facilitate comparison with previous reviews by Doll and Peto^3^ and Siemiatycki *et al*.^9^

### Review and data extraction

Two of us (NG and DL) independently reviewed data for all of the 120 agents classified in Group 1 through October 2017 in volumes 1–120 of the IARC Monographs to identify entries that met the criteria defined above. These determinations were reviewed by a third person (KS) and any discrepancies were resolved by discussion. For each included agent, we extracted data on the cancer sites for which the human evidence was classified as sufficient, where the classification was established on the basis of epidemiologic studies of workers, and where the occurrence of exposure in workers was documented in the monograph.

We also summarised agents across six broad classes adapted from Cogliano *et al* 12: chemicals; chemical mixtures; metals and metal compounds; airborne particles; airborne complex mixtures, and radiation and radionuclides. We grouped arsenic with the metals, although it is now considered to be a metalloid, to avoid creating of class containing a single agent.

Information on settings where occupational exposure is likely to occur, as described in the pertinent monograph, was extracted. Primary routes of exposure were also recorded for agents in categories other than radiation and radionuclides. If the monograph did not provide this information, we consulted other sources, most often the NIOSH Pocket Guide to Chemical Hazards.^14^

## Results and discussion

### Counting occupational carcinogens

**Figure 1:**
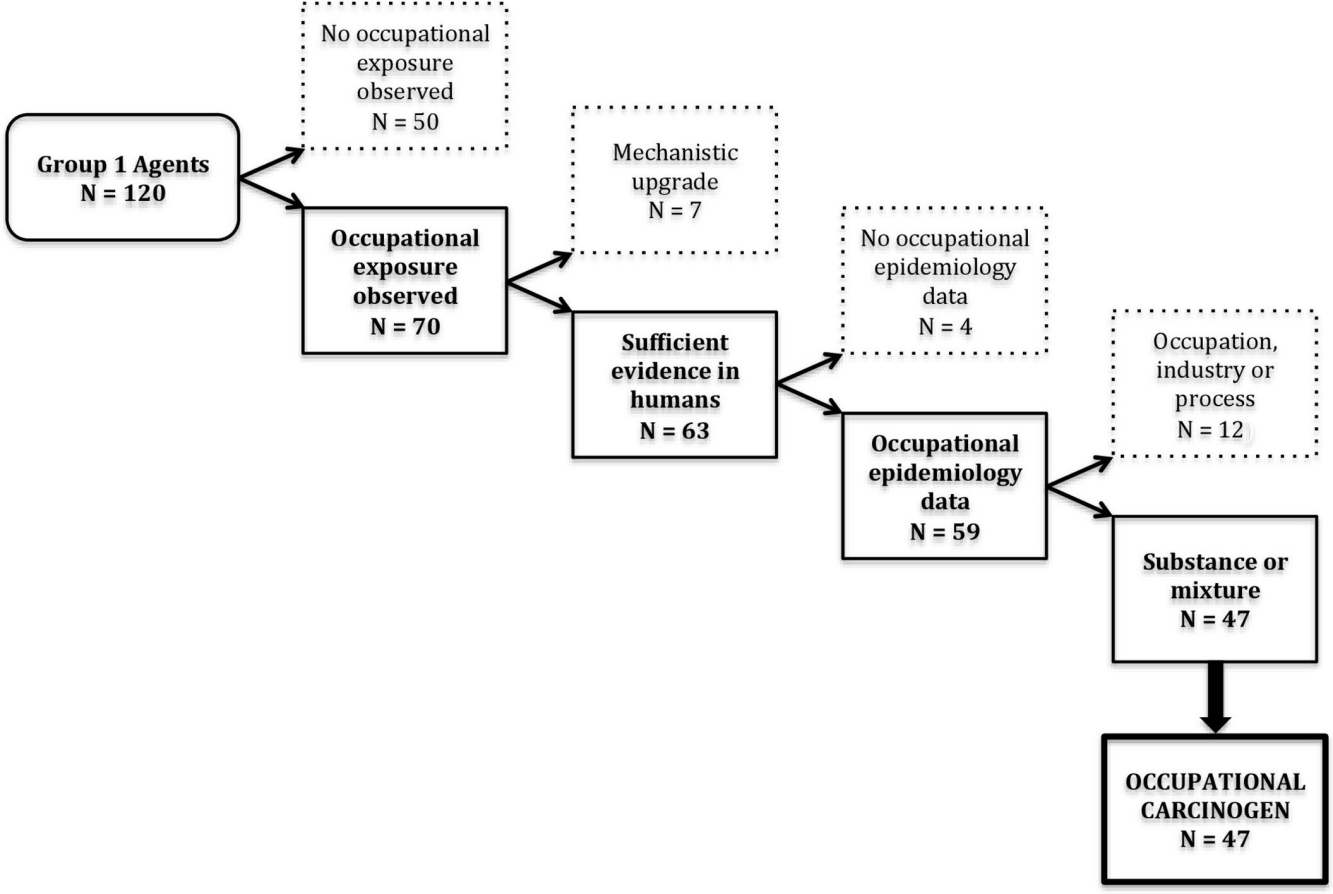
Defining occupational carcinogens from the International Agency for Research on Cancer (IARC) Monographs (1971–2017).

Among the 120 agents classified in IARC Group 1, 70 included mention of occupational exposures in the monographs (figure 1). Of these 70 Group 1 agents, 63 had sufficient evidence in humans (figure 1). The other seven had indications of occupational exposure but had been upgraded to Group 1 based on mechanistic evidence when the human evidence was less than ‘sufficient’. These agents were therefore excluded from our count of occupational carcinogens: ethylene oxide, dyes metabolised to benzidine, neutron radiation, benzo(a)pyrene, 2,3,4,7,8-pentachlorodibenzofuran, 4,4′-methylenebis(2-chloroaniline) and dioxin-like polychlorinated biphenyls.

Of the 63 Group 1 agents with ‘sufficient evidence of carcinogenicity’ in humans, 59 evaluations were based at least in part on studies of exposed workers (figure 1). The other four agents (aflatoxins, the asbestos-like fibres erionite and fluoroedenite and fission products including Strontium-90) were excluded since occurrence of occupational exposure was noted but no occupational epidemiology data were reported.

Among these 59 retained agents, 47 were individual substances, mixtures or types of radiation and 12 were occupations, industries or processes (figure 1). Although the IARC Monographs aim to identify and evaluate specific agents, some processes, industries and occupations have been classified in Group 1 with ‘sufficient evidence of carcinogenicity’ in humans (table 1). These evaluations were typically produced at a time when the available data provided a clear indication of increased cancer risk in an occupational group, but not enough information to identify a causal agent. While such broadly defined carcinogenic agents can lead to general industrial hygiene interventions, provide support to compensate exposed workers and stimulate research to identify specific causes, they have limited utility for informing specific prevention activities and may be affected by changes in processes, materials and exposure levels over time.

**Table 1:** Group 1 agents evaluated in the IARC Monographs Volumes 1–120, excluded from primary list of occupational carcinogens

Agent	Volume (a)	Year (a)	Cancers with sufficient evidence in humans (b)
*Reason for Exclusion: Group 1 classification based on mechanistic upgrade*			
Ethylene oxide	60	1994	N/A
2,3,4,7,8-Pentachlorodibenzofuran	100F	2012	N/A
3,4,5,3’,4’-Pentachlorobiphenyl (PCB-126)	100F	2012	N/A
4,4'-Methylenebis(2-chloroaniline) (MOCA)	99	2010	N/A
Benzidine, dyes metabolized to	99	2010	N/A
Benzo(*a*)pyrene	92	2010	N/A
Neutron radiation	75	2000	N/A
*Reason for Exclusion: Evaluation did not include occupational epidemiology data*			
Aflatoxins	Sup 7	1987	Liver
Erionite	Sup 7	1987	Mesothelioma
Fission products, including strontium-90	100D	2012	Salivary gland, oesophagus, stomach, colon, lung, bone, basal cell of the skin, female breast, kidney, urinary bladder, brain and CNS, thyroid, leukaemia
Fluoro-edenite fibrous amphibole	111	2017	Mesothelioma
*Reason for Exclusion: Group 1 classification is for an occupation, industry, or process*			
Acheson process, occupational exposure associated with	111	2017	Lung
Aluminium production	Sup 7	1987	Lung, bladder
Auramine production	Sup 7	1987	Bladder
Coal gasification	Sup 7	1987	Lung
Coal-tar distillation	92	2010	Skin
Coke production	Sup 7	1987	Lung
Haematite mining (underground, with exposure to radon)(c)	Sup 7	1987	Lung
Iron and steel founding (occupational exposure during)	Sup 7	1987	Lung
Isopropyl alcohol manufacture using strong acids	Sup 7	1987	Nasal cavity
Magenta production	Sup 7	1987	Bladder
Painter (occupational exposure as a)	47	1989	Lung, mesothelioma, bladder
Rubber manufacturing industry (occupational exposures in)	Sup 7	1987	Leukaemia, lymphoma, lung, stomach, bladder

The 47 specific substances, mixtures and types of radiation defined as occupational carcinogens are listed in table 2, with the cancer sites for which sufficient evidence was obtained.

**Table 2:** Occupational carcinogens evaluated in the IARC Monographs volumes 1–120 and comparison with two previous published listings

Agent	Volume†	Year†	Primary exposure routes‡			Human cancers with sufficient evidence§	Quantitative exposure- response data available	Included in Siemiatycki *et al* 9	Included in Doll and Peto^3^	Occupational exposure settings¶	Class
			Ingestion	Inhalation	Dermal contact						
1,2-Dichloropropane	110	2017		x		Biliary tract				Manufacture of plastic products, paints and other chemicals; printing; car painting	Chemicals
1,3-Butadiene	97	2008		x		Haematolymphatic organs	x			Manufacture of industrial chemicals, rubber products and plastic products; petroleum refining and petrochemical industries; building construction	Chemicals
2-Naphthylamine	4	1973		x	x	Urinary bladder		x	x	Manufacture of industrial chemicals and dyes	Chemicals
2,3,7,8-Tetrachlorodibenzo-*para*-dioxin	69	1997	x	x	x	All cancers combined	x	x		Manufacture of chemicals; herbicide handling and spraying; waste incineration	Chemicals
4-Aminobiphenyl	1	1972		x	x	Urinary bladder		x	x	Manufacture of chemicals and rubber	Chemicals
Acid mists, strong inorganic	54	1992	x	x	x	Larynx		x		Manufacture of soaps and detergents, phosphate fertilisers, lead batteries and other chemicals; electroplating and pickling	Airborne particles
Arsenic and inorganic arsenic compounds†	2	1973	x	x		Lung, skin, bladder	x	x	x	Manufacture of glass, pesticides and other chemicals; agricultural settings; mining, smelting and refining of metals; medical and veterinary procedures	Metals and metal compounds
Asbestos (all forms, including actinolite, amosite, anthophyllite, chrysotile, crocidolite, tremolite)	2	1973		x		Lung, mesothelioma, larynx, ovary	x	x	x	Mining, processing, transportation and handling of asbestos; work in shipyards; manufacture and use of asbestos-containing products	Airborne particles
Benzene	7	1974		x	x	Leukaemia (acute myeloid)	x	x	x	Manufacture and use of paints, rubber products, glues and other chemicals; distribution and handling of petrol; shoe manufacturing and repair	Chemicals
Benzidine	1	1972		x	x	Bladder		x	x	Manufacture of chemicals, dyes, rubbers and plastics	Chemicals
Beryllium and beryllium compounds	58	1993		x	x	Lung	x	x		Beryllium extraction, processing and fabrication; manufacture of electrical equipment, electronic components, aerospace materials; dental laboratory procedures	Metals and metal compounds
Bis(chloromethyl)ether; chloromethyl methyl ether (technical grade)	4	1974		x		Lung		x	Yes	Manufacture of chemicals; laboratory procedures	Chemicals
Cadmium and cadmium compounds	58	1993		x		Lung	x	x		Production, refining, and processing of cadmium and its alloys; manufacture of batteries and pigments	Metals and metal compounds
Chromium (VI) compounds	Sup 7	1987		x		Lung	x	x	Yes	Production and use of chromate pigments and paints; chrome plating; work in chrome-alloy foundries	Metals and metal compounds
Coal-tar pitch	35	1985		x	x	Lung, skin		x		Production of coal-tar products; roofing and surface coating activities	Chemical mixtures
Engine exhaust, diesel	105	2013		x		Lung	x			Rail, truck, and bus operation and mechanical maintenance; mining; firefighting	Airborne complex mixtures
Formaldehyde	88	2006		x	x	Nasopharynx, leukaemia	x			Manufacture of formaldehyde and other chemicals; histopathology and anatomy dissections; hospital disinfection; embalming	Chemicals
Ionising radiation (all types)**	100D	2012				None specified	x	x	x	Outdoor work involving sun exposure; nuclear fuel production and use; air travel; mining; human and veterinary medicine	Radiation and radionuclides
Leather dust	25	1981		x		Nasal cavity and paranasal sinus				Manufacture, processing and repair of leather, boots and shoes	Airborne particles
Lindane (see also hexachlorocyclohexanes)	113	2015*		x	x	Non-Hodgkin’s lymphoma	x			Manufacture of lindane; treatment of wood and wooden structures; agricultural application on livestock and crops	Chemicals
Mineral oils, untreated or mildly treated	3	1973		x	x	Skin		x		Paraffin processing; manufacture of metal products; metal working	Chemical mixtures
Nickel compounds	49	1990	x	x	x	Lung, nasal cavity and paranasal sinuses	x	x	x	Mining, smelting and refining of nickel; production of nickel alloys, stainless steel and batteries; electroplating; paint production and use	Metals and metal compounds
*ortho*-Toluidine	99	2010		x	x	Urinary bladder				Manufacture of *orth*o-toluidine and dyes, pigments, and some rubber chemicals; clinical and pathological laboratories	Chemicals
Outdoor air pollution**	109	2016		x		Lung	x			Where majority of working time is spent in polluted outdoor environments (eg, urban traffic police, professional drivers, street vendors)	Airborne particles
Particulate matter in outdoor air pollution	109	2016		x		Lung	x				Airborne particles
Pentachlorophenol	117	2016*		x	x	Non-Hodgkin’s lymphoma	x			Manufacture of PCP and other chemicals; agricultural settings; treatment of wood products; waste incineration	Chemicals
Plutonium	78	2001				Bone, liver, lung	x			Nuclear industry workers	Radiation and radionuclides
Polychlorinated biphenyls	107	2016		x	x	Malignant melanoma	x			Manufacture of PCB capacitors; manufacture and repair of transformers; waste incineration and recycling; firefighting	Chemical mixtures
Radioiodines, including iodine-131††	78	2001				Thyroid	x			Workers involved in nuclear accident clean-up	Radiation and radionuclides
Radionuclides, alpha-particle emitting, internally deposited**	78	2001				None specified	x			Mining and processing of uranium and other minerals; nuclear industry workers; human and veterinary medicine	Radiation and radionuclides
Radionuclides, beta-particle emitting, internally deposited**	78	2001				None specified	x				Radiation and radionuclides
Radium-224 and its decay products§	78	2001				Bone				Luminising industries	Radiation and radionuclides
Radium-226 and its decay products	78	2001				Bone, mastoid process, paranasal sinus					Radiation and radionuclides
Radium-228 and its decay products	78	2001				Bone, mastoid process, paranasal sinus					Radiation and radionuclides
Radon-222 and its decay products	43	1988				Lung	x			Mining and other underground work; mineral processing	Radiation and radionuclides
Shale oils	35	1985			x	Skin		x		Mining and production of shale oils and products; manufacturing of cotton textiles	Chemical mixtures
Silica dust, crystalline, in the form of quartz or cristobalite	68	1997		x		Lung	x	x		Mining and quarrying operations; foundries; ceramics, cement and glass industries; construction activities	Airborne particles
Solar radiation**	55	1992				Skin (basal cell carcinoma, squamous cell carcinoma, melanoma)	x			Outdoor work with sun exposure	Radiation and radionuclides
Soot	3	1973		x	x	Lung, skin		x	x	Industries and tasks with exposure to combustion products (eg, coke-making, chimney cleaning, incineration)	Airborne particles
Sulfur mustard (see also mustard gas)	9	1975		x		Lung		x	x	Manufacture of mustard gas; military service in WWI	Chemicals
Tobacco smoke, secondhand**	83	2004		x		Lung				Work in public settings where smoking occurs (eg, restaurants, bars, casinos, planes)	Airborne complex mixtures
Trichloroethylene	106	2014		x	x	Kidney				Manufacture of metals and plastic products; printing; textile furnishing; dry cleaning; construction	Chemicals
Ultraviolet radiation**	118	2017*				Eye, skin	x		x	Various work environments where welding is performed	Radiation and radionuclides
Vinyl chloride	7	1974		x		Liver (angiosarcoma, hepatocellular carcinoma)	x	x	x	Manufacture of polyvinyl chloride	Chemicals
Welding fumes	118	2017*		x		Lung	x			Various work environments where welding is performed	Airborne particles
Wood dust	62	1995		x		Nasal cavity and paranasal sinus, nasopharynx	x	x		Forestry and logging; sawmilling; manufacture of wood products; carpentry; construction	Airborne particles
X-radiation and gamma-radiation**	75	2000				Multiple, including: breast; leukaemia; thyroid; bone; brain and central nervous system; colon; kidney; lung; oesophagus; salivary gland; skin; stomach; bladder	x			Nuclear industry workers; human and veterinary medicine; workers involved in nuclear accident clean-up	Radiation and radionuclides

*Monographs still in press.

†Volume and year of publication correspond to the first instance of a Group 1 classification for the agent.

‡Routes not listed for radiations and radionuclides

§The cancer sites listed reflect the most recent IARC evaluation of the agent.

¶Examples of potentially exposed industries, work locations and/or occupations described in the relevant monograph; do not represent exhaustive summaries of past and present exposure scenarios.

**Occupational and non-occupational data contributed to first Group 1 evaluation.

††Occupational data contributed to subsequent Group 1 evaluation.

IARC, International Agency for Research on Cancer; PCB, polychlorinated biphenyl; PCP, pentachlorophenol; WWI, First World War.

Our working definition of an occupational carcinogen was developed with high specificity to ensure confidence that the observed associations between exposure and cancer were causal and substance specific. The number of occupational carcinogens estimated using these criteria consequently represents a lower limit. The definition of an occupational carcinogen could be expanded to include the 12 occupations and industries with sufficient evidence in humans, the seven agents with less than sufficient evidence of carcinogenicity in humans that were upgraded to Group 1 on mechanistic grounds, or the four agents with evidence of occupational exposure but no contributing data from occupational epidemiology studies. Similarly, occupational exposures to some biological agents and pharmaceuticals have been documented elsewhere in the literature, and those with sufficient evidence in humans could be considered as occupational carcinogens.

The number of carcinogens in the workplace may be substantially larger for additional reasons. New substances are introduced into workplace and environmental settings faster than information on potential health effects can be generated. For example, over 80 000 chemicals are currently registered for use in the USA alone, but only a small fraction have ever been evaluated for carcinogenicity.^15^ Because of limited resources, no carcinogen evaluation programme is able to evaluate all agents of potential interest. Accordingly, the IARC Monographs give higher priority to evaluating agents for which there are indications of human exposure and scientific data suggestive of carcinogenicity. Nevertheless, among the approximately 1000 agents IARC has evaluated, the evidence on cancer in humans has been judged to be inadequate for the majority. This determination is often reached when no relevant epidemiological studies have been done, the number of studies available is too small to be conclusive, the studies are of low quality, or the findings are inconsistent across studies.

### Cancer sites, agents and exposure routes

Twenty-three different types of cancer are causally associated with the 47 specific occupational carcinogens identified in this paper (table 3). Some cancers (eg, lung, urinary bladder, skin) are associated with multiple agents, and some agents are associated with more than one type of cancer. Among these, lung cancer was the most common, representing nearly a quarter (23%) of all agent-cancer associations. Other cancers that occurred frequently were skin cancer (10%), bone cancer (9%), bladder cancer (7%) and cancers of the nasal cavity and paranasal sinuses (6%) (table 3).

**Table 3:** Cancers caused by occupational carcinogens (n=47 agents), evaluated in IARC Monographs volumes 1–120

Cancers with sufficient evidence in humans	Agents	Number of occurrences	%
Lung	Bis(chloromethyl)ether; chloromethyl methyl ether (technical -grade); Coal-tar pitch; Sulfur mustard; Arsenic and inorganic arsenic compounds; Beryllium and beryllium compounds; Cadmium and cadmium compounds; Chromium (VI) compounds; Nickel compounds; Asbestos (all forms, including actinolite, amosite, anthophyllite, chrysotile, crocidolite, tremolite); Particulate matter in outdoor air pollution; Silica dust, crystalline, in the form of quartz or cristobalite; Soot; Welding fumes; Engine exhaust, diesel; Outdoor air pollution; Tobacco smoke, secondhand; X-radiation and Gamma-Radiation; Plutonium; Radon-222 and its decay products	19	23
Skin	Coal-tar pitch; Mineral oils, untreated or mildly treated; Shale oils; Arsenic and inorganic arsenic compounds; Soot; X-radiation and Gamma-Radiation; Solar radiation; Ultraviolet radiation	8	10
Bone, including mastoid process	X-radiation and Gamma-Radiation; Plutonium; Radium-224 and its decay products; Radium-226 and its decay products; Radium-226 and its decay products	5	6
Haematolymphatic system, including leukaemia, NHL	1,3-Butadiene; Benzene; Coal-tar pitch; X-radiation and Gamma-Radiation; Formaldehyde; Lindane; Pentachlorophenol	7	9
Leukaemia	Benzene; Coal-tar pitch; X-radiation and Gamma-Radiation	3	4
Non-Hodgkin lymphomanNon-Hodgkin’s lymphoma	Formaldehyde; Lindane; Pentachlorophenol	3	4
Urinary bladder	*ortho*-Toluidine; Arsenic and inorganic arsenic compounds; X-radiation and Gamma-Radiation; 2-Naphthylamine; 4-Aminobiphenyl; Benzidiene	6	7
Nasal cavity and paranasal sinus	Acid mists, strong inorganic; Chromium (VI) compounds; Leather dust; Nickel compounds; Wood dust	5	6
Thyroid	X-radiation and Gamma-Radiation; Radioiodines, including iodine-131	2	2
Breast	X-radiation and Gamma-Radiation	1	1
Kidney	Trichloroethylene; X-radiation and Gamma-Radiation	2	2
Larynx	Asbestos (all forms, including actinolite, amosite, anthophyllite, chrysotile, crocidolite, tremolite); Acid mists, strong inorganic	2	2
Liver	Plutonium; Vinyl chloride	2	2
Nasopharynx	Formaldehyde; Wood dust	2	2
All cancers combined	2,3,7,8-Tetrachlorodibenzo-*para*-dioxin	1	1
Biliary tract	1,2-Dichloropropane	1	1
Brain and central nervous system	X-radiation and Gamma-Radiation	1	1
Colon	X-radiation and Gamma-Radiation	1	1
EsophagusOesophagus	X-radiation and Gamma-Radiation	1	1
Eye	Ultraviolet radiation	1	1
Malignant melanoma	Polychlorinated biphenyls	1	1
Mesothelioma	Asbestos (all forms, including actinolite, amosite, anthophyllite, chrysotile, crocidolite, tremolite)	1	1
Ovary	Asbestos (all forms, including actinolite, amosite, anthophyllite, chrysotile, crocidolite, tremolite)	1	1
Salivary gland	X-radiation and Gamma-Radiation	1	1
Stomach	X-radiation and Gamma-Radiation	1	1
Total		82	100

IARC, International Agency for Research on Cancer; NHL, non-Hodgkin lymphoma.

While the patterns of frequently occurring cancers are clear, the exact numbers are subject to interpretation because the reporting of cancer sites in the monographs necessarily depends on the data available at the time of the evaluation. Some of the tumour sites listed in table 3 could justifiably be combined, resulting in higher counts for certain cancers, such as the aggregate of tumours of lymphatic and haematopoietic tissues (9%), but with a corresponding loss of detail. The number of cancer sites associated with an agent can also increase over time if new data become available. This was the case, for example, with asbestos: the original evaluation was based only on mesothelioma and lung cancer, but cancers of the larynx and ovary have been added in subsequent evaluations.^16^

**Figure 2:**
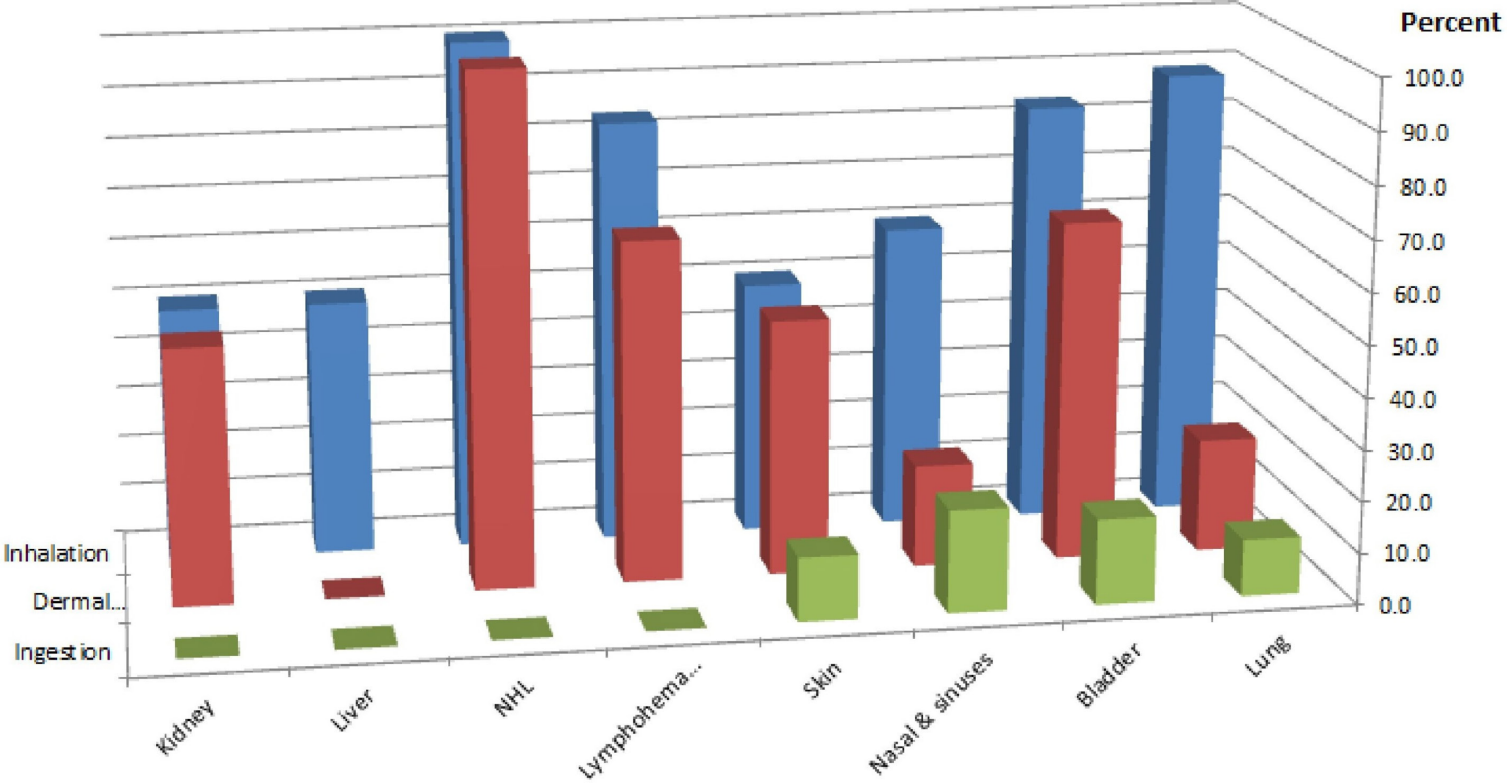
Route of exposure to occupational carcinogens and the cancers they cause (ionising radiation not included due to the diversity of exposure routes and cancer types). NHL, non-Hodgkin lymphoma.

Patterns relating the type of agent, routes of exposure and occurrence of cancer by organ site are also evident. Inhalation and skin absorption are the principal routes of exposure for most cancer sites (table 2). Not surprisingly, inhaled agents are associated primarily with lung, nasal and sinus cancers (figure 2).

Chemicals are associated with a diverse array of cancer sites, again with inhalation and skin absorption representing the principal routes of exposure to most (table 2). Cancers frequently associated with chemicals and chemical mixtures include tumours of the lymphohaematopoietic system (25%), bladder (20%), lung (15%) and skin (15%). The aggregate of cancers of the haematopoietic and lymphatic systems, including leukaemias and non-Hodgkin’s lymphomas, is mainly associated with exposure to chemicals through inhalation or contact with skin (table 2 and figure 2). To date, most chemicals are associated with only one cancer site, with the exception of formaldehyde, associated with leukaemia and cancer of the nasopharynx. Dioxin (2,3,7,8-tetrachlorodibenzo-*para*-dioxin) is unique in being associated most consistently with all cancers combined.^17^

Ionising radiation and radionuclides are associated with a wide array of different cancers (table 2), reflecting the varied physical properties and biological activities of these agents. X-radiation and gamma-radiation penetrate the whole body and are associated with numerous types of cancer, while radon (an inert gas) inhaled by underground miners causes lung cancer, and radium isotopes ingested by dial painters tend to be deposited in bones and teeth and are associated with cancer of bony tissues.^18^ Solar radiation and ultraviolet (UV) radiation are associated with several types of skin cancer (table 2). UV radiation generated in welding is also associated with cancer of the eye.^19^

We examined data for the 12 agents with ‘sufficient evidence of carcinogenicity’ for more than one cancer site, to identify cancers that tend to co-occur. Cancers of the lung and skin most often co-occurred together, due to exposure to coal-tar pitch, soot, arsenic and inorganic arsenic compounds. A similar examination of agents associated with cancers with both sufficient and limited evidence revealed combinations for cancers of the lung and bladder or kidney (data not shown). These patterns in cancers associated with exposure to certain carcinogens may be explained by route of exposure and physiochemical properties of the agents.

### Trends

A comparison of table 2 with previously published lists of occupational carcinogens suggests that progress continues to be made in identifying these agents despite the lack of adequate epidemiologic data for many occupational exposures. Furthermore, despite methodological differences in approach and changes in classification practices, the pace of identification appears to have increased over time. The list of 28 known occupational carcinogens developed by Siemiatycki *et al* 9 included 12 more agents than the list of 16 occupational carcinogens identified 23 years earlier by Doll and Peto.^3^ Table 2 of this paper includes 24 more agents added in the 14 years since Siemiatycki *et al* published their list.

Some methodological differences between reports are worth noting, however. Siemiatycki *et al* 9 combined all ‘ionizing radiation and sources thereof’ in a single listing and include talc-containing asbestiform fibres and erionite in their counts. In contrast, we list each type of ionising radiation separately, as in the monographs, and do not include asbestiform talc or erionite, as the former is classified with asbestos and the latter did not have occupational exposure documented in the monograph.

Although neither previous authors nor we included occupations, industries or processes in the final count of occupational carcinogens (note: Siemiatycki *et al* listed them in a separate table),^9^ it is noteworthy that the occurrence of such evaluations has declined over time. A few such Group 1 evaluations have been refined or superseded by evaluations of specific agents as improved exposure data have become available: the historical evaluation of ‘boot and shoe manufacturing and repair’ has been superseded by benzene and leather dust, ‘furniture and cabinet making’ has been replaced by wood dust and haematite mining has been made more specific by the addition of ‘underground, with exposure to radon.’ In contrast, only one new Group 1 classification of an occupation, industry or process (occupational exposures in the Acheson process for producing silicon carbide) has been added since 1989.

Improvements in the quality of epidemiologic studies may be a contributing factor in the increasing specificity of evaluations and the growth of knowledge about occupational carcinogens.^20^ Interest in identifying subtle risks, sometimes associated with low levels of exposure, has led to increasing emphasis on obtaining quantitative or semiquantitative exposure data. The presentation of exposure-response data can be taken as one marker of study quality because it requires collection of quantitative exposure data. Furthermore, analyses of exposure-response associations internal to an occupational cohort are also less susceptible to confounding and bias than comparisons to an external referent population. Exposure-response data were noted in the pertinent monographs for 29 occupational carcinogens (table 2), most from more recent evaluations from 2010 onwards. This trend may continue if efforts to collect and retain quantitative exposure data in occupational settings are successful.^21^

The growth and diversity of available scientific information may also contribute to the increasing numbers of occupational carcinogens identified. Bibliometric research shows that the number of published scientific articles, including medical and health sciences articles, has increased exponentially since Doll and Peto’s^3^ work was published.^22^ At the same time, science is becoming more global, with growing numbers of publications from outside the historical centres of Europe, the USA and Japan.^23^ Similar analyses of publications related to occupational health are not available, but statistics from the journal *Occupational & Environmental Medicine* suggest substantial growth and globalisation in this field, as well.^24^ Studies from diverse regions of the world are valuable for hazard identification, because they can support findings of causality by demonstrating consistency across populations and locations.^25^

There are signs for concern amid this growth, however. Some data indicate that since the 1990s, funding for occupational research has slowed or even declined in some high-income countries.^23 26^ Furthermore, significant gaps in knowledge remain concerning occupational exposures and diseases in low/middle-income countries where high exposures to many agents (which facilitate hazard identification) now tend to occur as a result of globalisation and the export of hazardous industries.^27 28^ For instance, in the People’s Republic of China, coke production increased more than fivefold between 1970 and 1995, while decreasing in Europe and North America.^29^ In several African countries, rapid developments in agricultural production have led to increased pesticide use, with implications for both occupational exposure and health.^30 31^

## Conclusions

Studies of workers have played a central role in identifying the causes of human cancer. Data compiled from the IARC Monographs from its initiation in 1971 through 2017 indicate that the number of recognised occupational carcinogens has increased progressively in recent decades. This trend may have been facilitated by advances in study quality, notably in quantitative exposure assessment, and in the global growth of the scientific literature base.

Despite notable progress, there continues to be a need for research on the causes of work-related cancer. Epidemiologic evidence is inadequate or entirely lacking for the majority of the over 1000 agents evaluated by IARC; many more agents present in workplaces have never been evaluated for carcinogenicity. There is also a need to identify the numbers of exposed workers by geographic location and to produce quantitative exposure data as a basis for hazard identification, exposure-response estimation and risk assessment.
